# PDGF in gliomas: more than just a growth factor?

**DOI:** 10.3109/03009734.2012.654860

**Published:** 2012-04-19

**Authors:** Nanna Lindberg, Eric C. Holland

**Affiliations:** Department of Neurosurgery, Department of Cancer Biology and Genetics, and Brain Tumor Center, 1275 York Ave, Memorial Sloan-Kettering Cancer Center, New York, NY 10021, USA

**Keywords:** Glioma, growth factor, oncogene, oncogenic stress, PDGF-B

## Abstract

Platelet-derived growth factor B (PDGF-B) is a growth factor promoting and regulating cell migration, proliferation, and differentiation, involved in both developmental processes and in maintaining tissue homeostasis under strict regulation. What are the implications of prolonged or uncontrolled growth factor signaling *in vivo,* and when does a growth factor such as PDGF-B become an oncogene? Under experimental conditions, PDGF-B induces proliferation and causes tumor induction. It is not known whether these tumors are strictly a PDGF-B-driven proliferation of cells or associated with secondary genetic events such as acquired mutations or methylation-mediated gene silencing promoting neoplasia. If PDGF-B-driven tumorigenesis was only cellular proliferation, associated changes in gene expression would thus be correlated with proliferation and not associated with secondary events involved in tumorigenesis and neoplastic transformation such as cycle delay, DNA damage response, and cell death. Changes in gene expression might be expected to be reversible, as is PDGF-B-driven proliferation under normal circumstances. Since PDGF signaling is involved in oligodendrocyte progenitor cell differentiation and maintenance, it is likely that PDGF-B stimulates proliferation of a pool of cells with that phenotype, and inhibition of PDGF-B signaling would result in reduced expression of oligodendrocyte-associated genes. More importantly, inhibition of PDGF signaling would be expected to result in reversion of genes induced by PDGF-B accompanied by a decrease in proliferation. However, if PDGF-B-driven tumorigenesis is more than simply a proliferation of cells, inhibition of PDGF signaling may not reverse gene expression or halt proliferation. These fundamental questions concerning PDGF-B as a potential oncogene have not been resolved.

## Introduction

### Platelet-derived growth factor B

Platelet-derived growth factor B (PDGF-B) was firstly isolated as a protein produced by platelets that stimulate DNA synthesis and growth of cells in culture, hence the name (1). The physiological function of this 30-kDa protein was not yet known except for its growth-promoting properties of cultured cells. PDGF-B binds as a dimer to cell surface receptors, upon which they are dimerized and phosphorylated. The auto-phosphorylation activates the receptors and enables phosphorylation of downstream targets by the intracellular receptor kinase domain, resulting in a signaling cascade, which ultimately promotes DNA synthesis and cell division in concert. This proliferation-promoting mechanism of growth factors and their receptors is essential for cells to multiply through embryogenesis and in a healthy organism. PDGF-B is also important in initiating cytoskeletal remodeling and cell motility in wound healing (2,3).

## What are growth factors?

Both growth factors and oncogenes stimulate the proliferation of cells. One definition of a growth factor is ‘a naturally occurring substance capable of stimulating cellular growth, proliferation, and cellular differentiation' (4). Perhaps an even better definition is ‘a complex family of polypeptide hormones or biological factors that are produced by the body to control growth, division, and maturation‘ (5). However, not all proteins classified as growth factors stimulate differentiation; some even promote dedifferentiation, such as PDGF-B (6,7), and some have dual roles stimulating proliferation and/or differentiation depending on the context, tissue, and cell type. Another example where a growth factor does not promote differentiation is epidermal growth factor (EGF), which stimulates proliferation of undifferentiated cell types without promoting differentiation to maintain a constant pool of stem cells in the brain (8,9). EGF is also important in maintaining undifferentiated pancreatic cells, and upon its removal cells differentiate (10). In the central nervous system, PDGF-B initially stimulates differentiation of undifferentiated glia to progenitor states, subsequently maintaining a pool of oligodendrocyte progenitor cells without promoting further differentiation (11–16). Growth factors stimulate differentiation, dedifferentiation, or maintenance of differentiation status in a context-dependent way that does not always end in terminal differentiation.

## Growth factor-regulated differentiation

Under normal circumstances, growth factor-driven differentiation results in differentiation accompanied by cell cycle exit. In most cases differentiation is terminal. However, there are experimental studies demonstrating plasticity in differentiation, where growth factors induce reversion to or maintain a less differentiated phenotype. PDGF-B can induce dedifferentiation of astrocytes into oligodendrocyte progenitor cells (6). Growth factors can also maintain less differentiated cells. In mammary tissue forced expression of TGF-beta inhibits growth and differentiation without any signs of inducing neoplasia, indicating that this growth factor is important in maintaining a pool of undifferentiated progenitor cells of the mammary tissue. This process of maintaining tissue homeostasis while preventing development of the mammary gland is also reversible where removal of the growth factor results in differentiation (17). This flexibility in growth factor-driven proliferation (and to some extent differentiation) indicates that arrested cells or terminally differentiated cells may still be susceptible to exogenous growth factor stimulation. Even differentiated mouse (18) and human cells (19–21) can be reprogrammed to dedifferentiate. However, it is likely that additional events are required for a growth factor to induce a malignant transformation and ultimately cancer.

## Roles of normal cellular proliferation and cell division

PDGF-B is a growth factor in that it regulates cellular growth, i.e. cell division. Indirectly that means the duplication of the DNA content of a cell co-ordinated with segregation of the chromosomes, production of cellular components and structural elements, and physical division of the cell into two daughter cells. Cell division can be symmetric or asymmetric, generating two identical daughter cells or two cells of different fate (22). The ratio of symmetric and asymmetric cell division can regulate the amount of a specific cell type or stem cell and their differentiated progeny to maintain tissue homeostasis. It is believed that stem cells self-renew by asymmetric division, generating one stem cell and one cell committed to differentiation. Cell division occurs naturally at a higher frequency during embryogenesis to build up tissue and organs. A temporary increase in growth factors promoting increased proliferation and/or differentiation to repair damage to tissue due to injury is also a normal function. Throughout life a constant pool of stem cells is maintained by asymmetric and symmetric proliferation. In case of injury or disease, stem cells can have a burst of proliferation to produce more differentiated cells in order to replace damaged or dead ones. Cellular proliferation also exists in tissues to make up for normal loss of cells; in highly proliferative tissues such as skin and mucosal membranes or bone-marrow this is a rapid process, and in other tissues such as brain or kidney it is almost negligible. Proliferation occurring under the aforementioned circumstances is normal and beneficial. These proliferative cues are under strict control of cellular mechanisms in place to prevent cells from inappropriate division generating a larger pool of cells than necessary or vice versa.

## Control of normal growth factor-driven proliferation

If production of cells exceeds what is necessary for a healthy organism it could be pathological. Having too many cells is deleterious to any organism since they require oxygen and nutrients. Excessive cells also need physical space, which can damage or impair the function of neighboring cells and the entire organ. These cells can also negatively influence surrounding cells by signaling through secreted factors or by direct cell–cell interactions. Such proliferations of cells may or may not be limited to cancer but is under any circumstance a pathological condition. In a normal cell or tissue, growth factor signaling most commonly leads to proliferation and ultimately differentiation and senescence, which functions as an anti-tumor barrier. One of the things that distinguish normal growth factor-driven cell proliferation from cancer is that cancerous cells evade senescence and continue to grow independently, possibly due to acquiring other genetic changes that promote cellular growth and inhibit senescence and/or apoptosis. Growth factor-driven proliferation is reversible in a normal cell: upon removal or inhibition of growth factor signaling a normal cell would arrest or undergo apoptosis (23–30). PDGF-B is known for its role in establishing the oligodendrocyte glial lineage and sustaining a pool of oligodendrocyte progenitor cells by signaling through the PDGF receptor alpha (PDGFRA). Infusion of PDGF-A in the ventricular system for several days can cause expansion of cells expressing the PDGFRA and induction of some glioma characteristics. However, this expansion is reversible upon removal of PDGF-A, indicating that this effect is a growth factor-driven proliferation of a subtype of cells responsive to PDGF signaling (31).

## Prolonged or abnormal growth factor signaling

A direct consequence of proliferation is replicative stress. In addition, by-products of cellular metabolism accompanying proliferation, such as free radicals, promote errors in DNA. The replication process is inherently error-prone, and while most errors are corrected it is estimated that uncorrected errors that persist could range from 1 × 10^-4^ to 1 × 10^-6^ mutations per gamete for a given gene. There are paramount proof-reading functions of the cell that detect and repair errors. In case of proof-reading failure, other programs are activated to eliminate cells with DNA copy error in order to protect the integrity of the organism. However, this is not a 100% fail-proof system, which means that proliferation can result in cells with DNA errors, a risk increasing with increased proliferative rate. If a specific DNA mutation affects a gene involved in cell proliferation or survival, this could through positive selection result in a rapidly increasing pool of abnormal cells, and increased proliferation could secondarily lead to accumulation of more DNA damage.

What is cancer if not abnormal proliferation of abnormal cells? In fact, 90% of cancers arise in epithelial surfaces from cells with a high proliferative rate (32). According to the National Cancer Institute (NCI), ‘cancer is a term used for diseases in which abnormal cells divide without control and are able to invade other tissues'. An oncogene could be defined as a factor that induces uncontrolled cell division and invasive ability. So if the amount of a growth factor is high enough could it lead to cancer, hence function as an oncogene? Growth factor-induced proliferation leading to an increasing number of cells would not mean that these cells are able to invade other tissues. However, considering that replicative stress leads to errors in DNA, where further growth-promoting mutations will be selected for, elevated growth factor signaling could eventually lead to cancer through secondary accumulated mutations involving genes that regulate contact inhibition and migration. These steps are described as classical hallmarks of cancer (33), and oncogenic stress causing chromosomal instability and evoking DNA damage response is well established (34,35).

There is compelling evidence that replicative stress caused by growth factors can ultimately result in secondary genetic changes leading to cancer progression. Preneoplastic lesions of skin, urinary, lung, colon, and breast cancer already have activated DNA damage check-points (34,36). In a model of melanoma, skin grafts were made hyperplastic by addition of growth factors, such as basic fibroblast growth factor, stem cell factor, and endothelin-3, over 4 weeks. These hyperplasias had activated DNA damage response in terms of 53BP1 foci, phosphorylated histone H2AX and Chk2, increased p53 levels, and apoptosis likely as a result of DNA damage response (36). PDGF-B over-expression has also been shown to induce DNA damage response, genomic instability and ploidy in glial cells, and hyperplastic lesions of the brain and subsequent gliomas (37). Over-expression of cyclin E, common in cancer and rapidly cycling cells, results in increases in Ser 15-phosphorylated p53, γ-H2AX, and Ser 966-phosphorylated cohesin SMC1, which are all targets of the DNA damage response kinases ATM and ATR (34). This points to the conclusion that over-expression of PDGF-B could cause replicative stress and ultimately result in tumorigenesis.

## PDGF as an oncogene

Studies of oncogenic retroviruses have identified numerous transforming genes with structural resemblance to human growth factors, leading to the hypothesis that human cancers may be caused by growth factors (38). In 1983 it was found that the active domain of PDGF-B shared an almost identical structure with the cancer-causing gene of Simian sarcoma virus, v-sis (39–41) and that there were multiple PDGF homologues (42,43). Further, many cancers harbor genetic alterations in growth factor-encoding genes (44–46), and brain tumors express the c-sis chain (PDGF-B) (47). Are these alterations cancer-causing or a result of cancer-associated hyperproliferation in cells that are positively selected for? In support, in a family with a germline missense mutation of PDGFRA (G2675T) the mutated allele segregated with gastrointestinal stromal tumor (GIST) incidence (48). Another PDGFRA germline mutation (V561D and Y555C) is also associated with sporadic GIST (49,50). Finally, in the case of gliomas, there have been at least two alterations identified in the PDGFR gene that lead to constitutive activation of the encoded protein (51).

Experimental studies have shown that PDGF can be causal in tumor formation *in vivo*. Retrovirus containing PDGF-B was introduced into newborn mice (52), causing fibrosarcoma, and later studies have confirmed that over-expression of PDGF-B can cause a variety of neoplasms, in particular brain tumors (53). One study indicated that there are additional events required for tumor progression upon PDGF-B stimulation (54), and concomitant loss of tumor suppressor genes increases malignancy in two viral models of PDGF-B-induced brain tumors (55,56). Additional events secondary to PDGF signaling might explain why growth factor inhibitors such as imatinib have had limited success in clinical trials so far (57,58); selection of patients with PDGFRA-positive glioblastomas did not confer any benefit of this treatment (59). Another possible reason why inhibitors of PDGF have had limited clinical success could be that chronic PDGF signaling leads to DNA methylation, which is not reversed by blocking receptor activity.

Classical oncogenes, like K-Ras, have aberrant signaling due to mutations rendering them more active. The most common form of mutated K-Ras, G12D, can induce and drive tumorigenesis, but in this case tumors are dependent on the oncogene and upon withdrawal they regress (60). This holds true for other oncogenes as well, such as H-Ras^V12G^ (61), MYC (62), and Bcr-Abl (63). However, when PDGF-B acts as an oncogene it is in its native form but is produced at higher levels, maybe in the wrong cell or at the wrong time. In this case the oncogenic effect of PDGF is in regulation or dysregulation of production of this otherwise normal protein. Removal of the signal might be insufficient, as indicated by the lack of effect of inhibitors of PDGF signaling, since neoplastic cells may have developed beyond dependence of PDGF-B signaling by acquiring other genetic or epigenetic modifications as a result of replicative stress. It is also possible that chronic PDGF-A stimulation induces glioma-like lesions that are reversible upon removal of PDGF-A (31) only if there are no additional events occurring. One study has demonstrated how PTK787-mediated inhibition of PDGF signaling in PDGF-B-induced gliomas results in a markedly reduced proliferation without elimination of tumors, indicating that these tumors are dependent on PDGF signaling to maintain proliferative capacity while inhibition does not eliminate tumors (64). The mechanism by which PDGF-B induces glioma, and if it is reversible, has yet to be determined.

## Is it possible to distinguish a growth factor from an oncogene?

Perhaps one could define an oncogene as something that induces proliferation of cells beyond what is reversible upon removal of that factor due to secondary events. Secondary events include genetic changes such as mutations, amplifications or deletions, and/or methylation-mediated gene silencing, resulting in changes of gene expression. A ‘normal' proliferation induced by a growth factor is reversible, where removal of the growth factor results in halted proliferation and reversion of gene expression changes associated with growth factor-driven proliferation. Is PDGF-B a growth factor, i.e. is PDGF-B-driven proliferation and glioma formation reversible, or is PDGF-B an oncogene?

One way of addressing this question experimentally would be to determine which changes in gene expression are induced by PDGF-B over-expression and if there is any proof of replicative stress or DNA methylation caused by chronic PDGF-B stimulation that could promote tumorigenesis. If PDGF-B is just a growth factor, changes in gene expression between PDGF-B-driven tumor cells and their normal counterpart, oligodendrocyte progenitor cells (OPCs), would be limited to genes involved in proliferation, i.e. cell cycle and cell division. However, if chronic PDGF-B stimulation causes replicative stress that could result in secondary genetic effects driving tumorigenesis, an increase in expression of genes involved in cell cycle delay and arrest, DNA damage response and possibly senescence or cell death would be seen. PDGF-B-driven proliferation in a normal cell is tightly regulated and reversible; upon growth factor removal proliferation is halted. By inhibiting PDGF signaling using a small molecule kinase receptor inhibitor a benign PDGF-B-driven proliferation is arrested and gene expression would return to that of an unstimulated cell, while a cell turned neoplastic would be less likely to respond. These experiments could be done using an *in-vivo* system where PDGF-B-driven proliferation is inhibited chemically and gene expression compared between normal OPCs, PDGF-B-stimulated cells, and cells treated with the small molecule kinase inhibitor. An experiment of that kind would answer the question if PDGF-B-driven proliferation is reversible and what gene expression changes are associated with PDGF-B.

The RCAS-TVA model has been used successfully to study PDGF-B-driven tumorigenesis *in vivo*. By using this model it is possible to induce expression of PDGF-B in specific subsets of glial cells *in vivo* by retroviral-mediated gene transfer. The Nestin-tva (Ntv-a) transgenic mice in an *Ink4a-Arf^-/-^ Pten^fl/fl^* background develop Olig2-positive glioblastomas upon PDGF-B expression homologous to human glioblastomas of the proneural subtype with the same histopathology, including pseudopalisading necrosis, microvascular proliferation, and high mitotic activity (65,66). This model also shares the genetic hallmarks of human GBMs of proneural subtype where PDGFRA, PTEN, and CDKN2A each are altered in 10%–70% of cases (67). Tumors are believed to originate from a glial progenitor cell and are almost ubiquitously positive for the basic helix-loop-helix transcription factor Olig2, which regulates the fate of neural and glial progenitor cells and promotes differentiation of the oligodendrocytic lineage. By combining the RCAS-TVA model with a reporter that would allow isolation of Olig2-expressing cells for expression analysis, one should be able to determine if the changes in expression that accompany oncogenic transformation of this cell population are reversed by blockade of PDGFR signaling. If all the expression changes were reversed by PDGFR blockade then one would argue that the effect of PDGF in this context was a growth factor. However, if only a minority or none of the changes induced by PDGF-induced gliomagenesis were reversed by PDGFR blockade one would then argue that PDGF was acting as an oncogene.
